# The use of antibiotics in the treatment of pediatric varicella patients: real-world evidence from the multi-country MARVEL study in Latin America & Europe

**DOI:** 10.1186/s12889-019-7071-z

**Published:** 2019-06-26

**Authors:** Lara J. Wolfson, Marìa Esther Castillo, Norberto Giglio, Zsófia Mészner, Zsuzsanna Molnár, Mirella Vàzquez, Jacek Wysocki, Alexandra Altland, Barbara J. Kuter, Melissa Stutz, Emmanouil Rampakakis, Craig S. Roberts

**Affiliations:** 1 Merck & Co., Inc., Center for Observational and Real-World Evidence (CORE), Kenilworth, NJ USA; 2 Instituto Nacional de Salud del Niño (INSN), Breña, Perú; 3 Universidad Peruana Cayetano Heredia, Lima, Perú; 4 Hospital de Niños Ricardo Gutiérrez, Buenos Aires, Argentina; 5 St. László Hospital for Infectious Diseases, National Institute of Child Health, Budapest, Hungary; 6 National Center for Epidemiology, Budapest, Hungary; 7 Instituto Nacional de Pediatría, Ciudad de México, México; 8 Poznan University of Medical Sciences, Poznan, Poland; 9 Merck & Co., Inc., Global Vaccines Medical Affairs, Kenilworth, NJ USA; 10 JSS Medical Research, Montréal, Québec Canada; 11 Department of Pediatrics, McGill University, Montréal, Québec Canada

**Keywords:** Varicella, Pediatrics, Antimicrobial stewardship, Observation study

## Abstract

**Background:**

Varicella is a highly contagious childhood disease. Generally benign, serious complications necessitating antibiotic use may occur. The objective of this study was to characterize the rate, appropriateness and patterns of real-world antibiotic prescribing for management of varicella-associated complications, prior to universal varicella vaccination (UVV) implementation.

**Methods:**

Pooled, post-hoc analysis of 5 international, multicenter, retrospective chart reviews studies (Argentina, Hungary, Mexico, Peru, Poland). Inpatient and outpatient primary pediatric (1–14 years) varicella cases, diagnosed between 2009 and 2016, were eligible. Outcomes, assessed descriptively, included varicella-associated complications and antibiotic use. Three antibiotic prescribing scenarios were defined based on complication profile in chart: evidence of microbiologically confirmed bacterial infection (Scenario A); insufficient evidence confirming microbiological confirmation (Scenario B); no evidence of microbiological confirmation (Scenario C). Stratification was performed by patient status (inpatient vs. outpatient) and country.

**Results:**

Four hundred one outpatients and 386 inpatients were included. Mean (SD) outpatient age was 3.6 (2.8) years; inpatient age was 3.1 (2.8) years. Male gender was predominant. Overall, 12.2% outpatients reported ≥1 infectious complication, 3.7% ≥1 bacterial infection, and 0.5% ≥1 microbiologically confirmed infection; inpatient complication rates were 78.8, 33.2 and 16.6%, respectively. Antibiotics were prescribed to 12.7% of outpatients and 68.9% of inpatients. Among users, β-lactamases (class), and clindamycin (agent), dominated prescriptions. Scenario A was assigned to 3.9% (outpatients) vs 13.2% (inpatients); Scenario B: 2.0% vs. 6.0%; Scenario C: 94.1% vs. 80.8%.

**Conclusions:**

High rates of infectious complications and antibiotic use are reported, with low rates of microbiological confirmation suggesting possible antibiotic misuse for management of varicella complications.

**Electronic supplementary material:**

The online version of this article (10.1186/s12889-019-7071-z) contains supplementary material, which is available to authorized users.

## Background

Varicella zoster virus (VZV) is a highly contagious disease infecting between 2 and 16 per 1000 individuals annually worldwide [1–3]. Generally, a childhood affliction, regional variations in incidence rates and age distribution are attributed to factors such as population density, climate, and earlier preschool/out-of-home care in children [4, 5]. Primary symptoms include appearance of a characteristic pruritic vesicular rash, as well as fever, malaise, anorexia, headache, and abdominal pain, occurring either concurrently, or 1–2 days before rash appearance [6].

Although usually following a benign course of disease, varicella can result in serious complications. In pediatric inpatient populations, the most commonly observed are skin and soft tissue infections, and neurological complications [7]. In addition, up to 28% of varicella outpatients have been found to report complications [8], with skin and soft tissue infections accounting for up to 90% of these [8]. Consequently, treatment of varicella-related complications in both inpatient and outpatient settings may involve use of antibiotic agents.

Recently, concerns over antimicrobial resistance have increased [9]. Proposed efforts to address resistance include antibiotic stewardship programs, aimed at improving antibiotic use to conserve effectiveness and reduce emergence of resistant strains, and use of vaccines to prevent infections that may result in antibiotic use [9, 10]. In this context, the purpose of this analysis is to characterize the rate, appropriateness and patterns of antibiotic use for management of varicella-associated complications in real-world clinical settings in the absence of universal varicella vaccination.

## Methods

### Study design and patient selection

This was a post-hoc analysis of 5 multicenter, retrospective chart review studies conducted as part of MARVEL (Multi-country economic and epidemiological burden of varicella). Burden of illness associated with varicella in patients ≤14 years of age was evaluated in Argentina (2009–2014) [8], Hungary (2011–2015) [11], Mexico (2011–2016) [12], Peru (2011–2016) (Castillo M, et al. Economic burden of varicella in children in peru, 2011-2016, Forthcoming), and Poland (2010–2015) [13]. Each study was approved by local ethics committees, and conducted according to local laws and regulations, as well in accordance with the Guidelines for Good Pharmacoepidemiology Practices (GPP).

Eligible patients were inpatients and outpatients with a primary varicella diagnosis by the pediatrician indicated in their patient chart. Outpatients were those who visited the doctor’s office, outpatient clinic/department of hospital, or emergency department (ER) without hospitalization for varicella. Inpatients were defined as those admitted to a hospital for primary varicella, including those initially seen in an outpatient setting.

Patients, in an approximate 1:1 ratio of outpatients to inpatients, were identified by investigators, who screened patient charts in their practices from the most recent year to 5 years previously until the target sample size was reached. The date of the first varicella report was identified, and each chart was reviewed from this date until resolution of disease, or date of last contact, if the resolution date was unavailable. Patients were excluded if they received prior varicella vaccination, presented with a second case of varicella, or had herpes zoster.

### Outcome measures

Sociodemographic characteristics, medical history, as well as disease parameters were extracted from patient charts. Varicella-related clinical complications were identified and profiled by complication type (clinically diagnosed as infectious, non-infectious, or “missing”), infection type (clinically diagnosed as bacterial, non- bacterial [viral, fungal or “other” specified], or missing), and microbiological confirmation (yes/no).

Antibiotics use, defined as type, dose, and duration of use, were obtained from the patient chart from the date of onset to the date of resolution or last patient contact.

### Statistical methods

All analyses were reported for the total cohort, stratified by patient status (outpatient vs. inpatient), and country. Descriptive statistics were produced for all study variables [mean, standard deviation (SD), 95% confidence interval (CI) of the mean] for continuous variables, and number and percentage for categorical variables.

Distribution of varicella complications was assessed descriptively as the number and proportion of patients with ≥1 clinically diagnosed infectious complication, ≥1 clinically diagnosed bacterial infection, and ≥ 1 microbiologically confirmed bacterial infection. Proportions by complication type were reported for overall clinically diagnosed infectious complications. All “other” complications were coded using the Medical Dictionary for Regulatory Activities, version 18.0/19.0, and were reported by system organ class.

The number and proportion of patients reporting use of ≥1 antibiotic was ascertained. Among antibiotic users, the mean number of antibiotics prescribed and duration of use (days), was assessed. Additionally, the number and proportion of patients prescribed ≥1 antibiotic by class and top 5 agents (within the pooled cohort) was evaluated.

Data collected did not allow for a direct link between infection type and antibiotic prescribed. Instead, antibiotic users were classified as having received antibiotics under 4 prescribing scenarios based on their complication profile present in the patient chart (see Additional file 1: Table S1 for the classification algorithm):

- **Scenario A:** evidence of a confirmed bacterial infection (clinically diagnosed and microbiologically confirmed)
- **Scenario B**: insufficient evidence to confirm/refute bacterial infection
- **Scenario C:** antibiotic prescribed without information confirming a bacterial infection
- **Scenario D:** potentially redundant antibiotic use, defined as number of antibiotics prescribed greater than the number of infectious complications.

The relationship between the number and proportion of antibiotic users by prescribing scenarios was reported to understand the potential inappropriate use of antibiotics (scenarios B, C and D). In addition, for patients classified according to Scenario C and Scenario B/C, the number and proportion reporting ≥1 antibiotic by class and agent was assessed.

Cumulative number of days of annual VZV-related antibiotic use was calculated based on previous estimates for the number of inpatient and outpatient cases of varicella by country [8, 11–13], as well as the proportion of patients administered antibiotics, and the corresponding mean duration of use, calculated as per described above.

Statistical analyses were performed using SAS® software version 9.4 (SAS Institute Inc., Cary, NC, USA).

## Results

### Baseline patient and disease characteristics

The total cohort included 787 patients (401 outpatients and 386 inpatients), distributed across the following locations: Argentina (*n* = 150, 19.1%); Hungary (*n* = 156, 19.8%); Mexico (*n* = 152 (19.3%); Peru (*n* = 179, 22.7%) and Poland (n = 150; 19.1%). Mean (SD) age in the total outpatient population was 3.6 (2.8) years; inpatients were found to be slightly younger that outpatients [mean (SD): 3.1 (2.8) years], and included more males (50.6% outpatients, 56.7%, inpatients) (Table 1).

**Table 1:** Baseline patient and disease characteristics by patient status and country

	Outpatients						Inpatients					
	Argentina	Hungary	Mexico	Peru	Poland	Total	Argentina	Hungary	Mexico	Peru	Poland	Total
	*N* = 75	*N* = 75	*N* = 75	*N* = 101	*N* = 75	*N* = 401	*N* = 75	*N* = 81	*N* = 77	*N* = 78	*N* = 75	*N* = 386
Gender, male, n (%)	40 (53.3)	33 (44.0)	31 (41.3)	58 (57.4)	41 (54.7)	203 (50.6)	46 (61.3)	45 (55.6)	39 (50.6)	43 (55.1)	46 (61.3)	219 (56.7)
Age, years, mean (SD)	3.8 (2.4)	4.4 (2.0)	3.0 (3.2)	3.3 (3.3)	3.9 (2.6)	3.6 (2.8)	2.9 (2.2)	3.7 (2.1)	2.6 (3.5)	2.4 (3.4)	4.2 (2.3)	3.1 (2.8)
Race, n (%)												
*Caucasian*	4 (5.3)	75 (100)	2 (2.7)	–	75 (100)	156 (38.9)	7 (9.3)	81 (100)	3 (3.9)	2 (2.6)	75 (100)	168 (43.5)
*Hispanic/Latino*	71 (94.7)	–	61 (81.3)	90 (89.1)	–	222 (55.4)	68 (90.7)	–	69 (89.6)	46 (59.0)	–	183 (47.4)
*Latino/Mestizo/Indigenous*	–	–	12 (16.0)	11 (10.9)	–	23 (5.7)	–	–	5 (6.5)	30 (38.5)	–	35 (9.1)
Area of residence, n (%)												
*Rural*	4 (5.3)	4 (5.3)	13 (17.3)	4 (4.0)	7 (9.3)	32 (8.0)	12 (16.0)	28 (34.6)	16 (20.8)	23 (29.5)	23 (30.7)	102 (26.4)
*Urban*	70 (93.3)	71 (94.7)	62 (82.7)	97 (96.0)	64 (85.3)	364 (90.8)	62 (82.7)	53 (65.4)	61 (79.2)	55 (70.5)	51 (68.0)	282 (73.1)
*Not available*	1 (1.3)	–	–	–	4 (5.3)	5 (1.2)	1 (1.3)	–	–	–	1 (1.3)	2 (0.5)
Maximum number of skin lesions, n (%)												
*< 50*	20 (26.7)	51 (68.0)	27 (36.0)	56 (55.4)	25 (33.3)	179 (44.6)	3 (4.0)	2 (2.5)	20 (26.0)	1 (1.3)	18 (24.0)	44 (11.4)
*50–249*	36 (48.0)	16 (21.3)	44 (58.7)	35 (34.7)	36 (48.0)	167 (41.6)	50 (66.7)	59 (72.8)	45 (58.4)	7 (9.0)	37 (49.3)	198 (51.3)
*250–500*	17 (22.7)	7 (9.3)	4 (5.3)	9 (8.9)	14 (18.7)	51 (12.7)	14 (18.7)	20 (24.7)	11 (14.3)	50 (64.1)	12 (16.0)	107 (27.7)
*> 500*	2 (2.7)	1 (1.3)	–	1 (1.0)	–	4 (1.0)	8 (10.7)	–	1 (1.3)	20 (25.6)	8 (10.7)	37 (9.6)
Immuno-compromised, yes, n (%)^a^	1 (1.3)	–	1 (1.3)	–	–	2 (0.5)	1 (1.3)	4 (4.9)	5 (6.5)	1 (1.3)	–	11 (2.8)

*SD* Standard Deviation

^a^Patients were considered immunocompromised if they had ≥1 of the following conditions: HIV/AIDS, congenital immunodeficiency, received systemic steroids, or had any other immunocompromised condition listed in their medical history

Based on the maximum number of skin lesions reported, there was more severe disease among inpatients, with over 35% reporting ≥250 lesions, compared to < 15% of outpatients. A compromised immune system was identified for 11 inpatients (2.8%) and 2 outpatients (0.5%) (Table 1).

### Varicella-associated complications

**Fig. 1:**
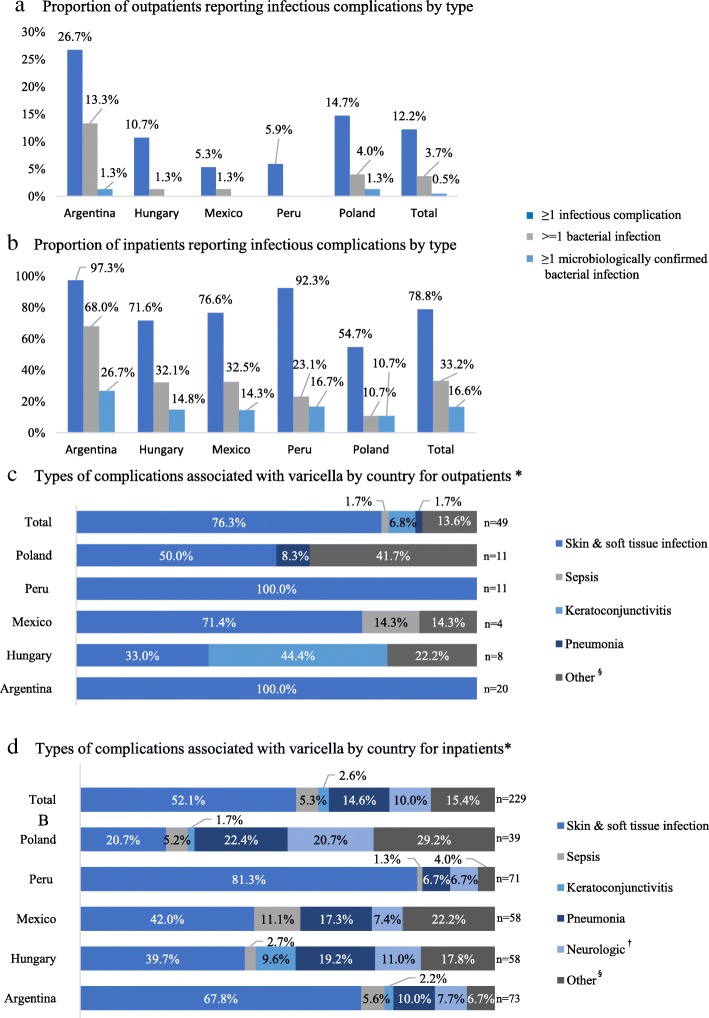
Infectious complications by patient status and country. * Proportions based on total number of infectious complications; patients may have reported ≥1 infectious complication. ** One outpatient was diagnosed with sepsis and died in the ER due to varicella-related toxic shock without hospital admission. ^§^ Other includes: nephritis, meningitis, hepatitis, acute osteomyelitis, septic arthritis and “other” complication categories. ^†^ Neurologic includes: encephalitis and cerebellitis

In the combined analysis, 12.2% of outpatients reported ≥1 infectious complication, of which 3.7% were identified as bacterial, and a further 0.5% were microbiologically confirmed bacterial infections (Fig. 1a). Inpatient rates reported for ≥1 infectious, bacterial, and microbiologically confirmed bacterial infections were 78.8, 33.2 and 16.6%, respectively (Fig. 1b). The highest proportion of complications was observed in Argentina (Fig. 1 a and b).

Skin and soft tissue infection was found to be the most common varicella-related complication accounting for 76.3% of outpatient (Fig. 1c) and 52.1% of inpatient (Fig. 1d) infectious complications, respectively, followed by sepsis (outpatients: 1.7%, inpatients: 5.3%), keratoconjunctivitis (6.8%, 2.6%), pneumonia (1.7%, 14.6%), neurologic (10.0%; inpatients only), and “other” complications (13.6%, 15.4%).

### Antibiotic use

The proportion reporting use of ≥1 antibiotic was 12.7% (*n* = 51/401) among outpatients and 68.9% (*n* = 266/386) among inpatients (Table 2). Mean (95% CI) number of antibiotics prescribed among users was highest in Latin American inpatients [Argentina: 2.1 (1.7, 2.5); Mexico: 2.5 (2.1, 2.9); Peru: 2.2 (1.8, 2.5)], as was duration of antibiotic use [10.6 (9.7, 11.5); 16.1 (15.0, 17.1); 14.6 (13.7, 15.5) days, respectively]. Overall outpatient [mean (95% CI)] estimates were lower for both number of antibiotics prescribed [1.4 (1.1, 1.7)] and duration of use [7.9 (6.9, 10.5)] days (Table 2).

**Table 2:** Patients reporting ≥1 antibiotic by class and mean duration of antibiotic use, among antibiotic users by patient status and country

	Outpatient						Inpatient					
	Argentina *N* = 75	Hungary *N* = 75	Mexico *N* = 75	Peru *N* = 101	Poland *N* = 75	Total *N* = 401	Argentina *N* = 75	Hungary *N* = 81	Mexico *N* = 77	Peru *N* = 78	Poland *N* = 75	Total *N* = 386
Patients prescribed ≥1 antibiotic, n (%)	17 (22.7)	2 (2.7)	6 (8.0)	17 (16.8)	9 (12.0)	51 (12.7)	54 (72.0)	45 (55.6)	57 (74.0)	67 (85.9)	43 (57.3)	266 (68.9)
Mean (95% CI), number of antibiotics prescribed per patient ^a^	1.8 (1.2, 2.5)	1.0 (0.2, 3.1)	1.5 (0.7, 2.7)	1.1 (0.7, 1.7)	1.1 (0.6, 1.9)	1.4 (1.1, 1.7)	2.1 (1.7, 2.5)	1.7 (1.4, 2.1)	2.5 (2.1, 2.9)	2.2 (1.8, 2.5)	1.5 (1.2, 1.9)	2.0 (1.9, 2.2)
Mean (95% CI) duration of antibiotics prescribed per patient, days ^a^	9.5 (8.1, 11.1)	7.0 (4.1, 11.8)	8.2 (6.2, 10.8))	6.5 (5.3, 7.8)	8.7 (6.9, 10.7)	7.9 (6.9, 10.5)	10.6 (9.7, 11.5)	7.2 (6.4, 8.0)	16.1 (15.0, 17.1)	14.6 (13.7, 15.5)	8.6 (7.7, 9.5)	10.9 (10.5, 11.3)
Patients prescribed ≥1 antibiotic, by class, n (%) ^a,b, c^												
*β-lactam agent*	15 (88.2)	2 (100.0)	4 (66.7)	12 (70.6)	8 (88.9)	41 (80.4)	32 (59.3)	43 (95.6)	51 (89.5)	66 (98.5)	37 (86.0)	229 (86.1)
*Lincosamide*	7 (41.2)	–	3 (50.0)	–	–	10 (19.6)	39 (72.2)	8 (17.8)	35 (61.4)	31 (46.3)	4 (9.3)	117 (44.0)
*Aminoglycide*	–	–	–	1 (5.9)	–	1 (2.0)	5 (9.3)	6 (13.3)	5 (8.8)	2 (3.0)	3 (7.0)	21 (7.9)
*Glycopeptide*	–	–	–	–	–	–	3 (5.6)	–	9 (15.8)	5 (7.5)	1 (2.3)	18 (6.8)
*Other*	7 (41.2)	–	1 (16.7)	5 (29.4)	1 (11.1)	14 (27.5)	12 (22.2)	–	2 (3.5)	1 (1.5)	–	15 (5.6)
*Macrolide*	–	–	–	1 (5.9)	1 (11.1)	2 (3.9)	–	–	2 (3.5)	–	5 (11.6)	7 (2.6)
*Fluoroquinolone*	–	–	–	–	–	–	–	1 (2.2)	2 (3.5)	–	1 (2.3)	4 (1.5)
Mean (95% CI) duration of use, by class, days ^a^												
*β-lactam agent*	5.8 (4.7, 7.1)	7.0 (4.1, 11.8)	4.3 (2.6, 6.8)	6.2 (4.9, 7.7)	7.1 (5.5, 9.2)	6.0 (5.1, 7.0)	8.4 (7.5, 9.5)	6.2 (5.5,7.0)	9.2 (8.4, 10.0)	10.1 (9.3, 10.9)	7.4 (6.5,8.3)	8.1 (7.8, 8.5)
*Lincosamide*	4.9 (3.5, 6.8)	–	6.0 (3.8, 9.5)		–	5.4 (4.1, 7.2)	5.4 (4.7, 6.1)	4.0 (2.8, 5.7)	8.1 (7.2, 9.1)	6.8 (5.9, 7.8)	8.5 (6.1, 11.9)	6.3 (5.7, 7.0)
*Aminoglycide*	–	–	–	1.0 (0.1, 7.1)	–	1.0 (0.1, 7.1)	4.2 (2.7, 6.4)	3.5 (2.3, 5.4)	5.4 (3.7, 7.8)	8.5 (5.3, 13.7)	5.7 (3.5, 9.1)	5.2 (4.3, 6.3)
*Glycopeptide*	–	–	–	–	–	–	6.0 (3.8, 9.5)	–	9.0 (7.2, 11.2)	14.8 (11.8, 18.6)	5.0 (NA)	8.0 (6.1, 10.3)
*Other*	8.7 (5.9, 12.7)	–	14.0 (8.3, 23.6)	5.6 (3.9, 8.1)	7.0 (3.3, 14.7)	8.3 (6.4, 10.8)	4.1 (3.1, 54)	–	6.3 (4.0, 9.9)	10.0(NA)	–	6.4 (4.9, 8.4)
*Macrolide*	–	–	–	7.0 (3.3, 14.7)	14.0 (8.3, 23.7)	9.9 (6.3, 15.6)	–	–	4.5 (2.3, 8.6)	–	6.6 (4.7, 9.3)	5.4 (3.8, 7.9)
*Fluoroquinolone*	–	–	–	–	–	–	–	3.0 (1.0, 9.3)	9.0 (5.7, 14.3)	–	7.0 (3.3, 14.7)	5.7 (3.6, 9.2)

*CI* Confidence Interval

^a^ Among population of antibiotic users

^b^ Reported for classes of antibiotics administered to > 1.0% of the total inpatient and/or outpatient population

^c^ Patients may have been prescribed ≥1 antibiotic

Among antibiotic users, prescriptions by antibiotic class involved predominantly β-lactam agents for all countries, with the exception of Argentinian inpatients, where 72.2% were administered lincosamide class antibiotics (*n* = 39/54). Generally, lincosamides were 2nd after β-lactam agents in terms of frequency of use in inpatients (Table 2). Overall mean (95% CI) duration of use of β-lactam agents was 6.0 (5.1, 7.0) days for outpatients, and 8.1 (7.8, 8.5) days for inpatients; lincosamide duration of use was slightly lower at 5.4 (4.1, 7.2) days and 6.3 (5.7, 7.0) days for outpatients and inpatients, respectively.

The top 5 antibiotic agents identified among users were clindamycin (19.6% outpatients; 44.0% inpatients); ceftriaxone (13.7%; 16.9%), cefuroxime (3.9%; 15.8%), penicillin (11.8%; 14.7%) and cefalexin (11.8%; 14.7%); both Hungarian and Polish inpatients, however, reported cefuroxime as the leading antibiotic agent prescribed, [24.4% (*n* = 11/45), 53.5% (=23/43)], respectively] (Additional file 2: Table S2). Highest outpatient duration of use was for cefalexin [7.1 (4.7, 10.6) days], whereas highest inpatient duration was for penicillin [6.7 (5.8, 7.7) days].

Extrapolated to the annual number of VZV-related pediatric inpatients and outpatients per country, cumulative duration of VZV-related antibiotic use per year was lowest in Hungary and Mexico at 77, 871 and 287,528 days, respectively, and highest in Peru (~ 1.6 million days) Poland (~ 1.8 million days,) and Argentina (~ 2.6 million days) (Additional file 3: Table S3).

### Prescribing scenarios

**Fig. 2:**
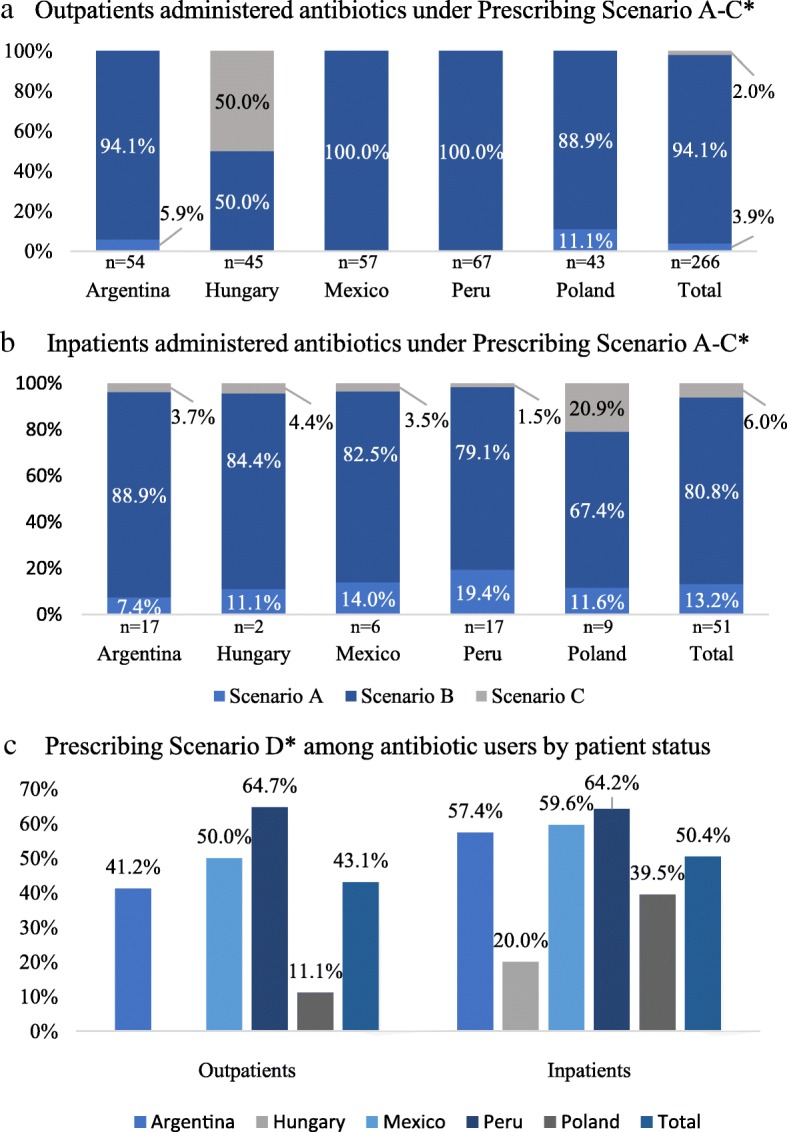
Antibiotic prescribing scenarios by patient status and country. *Prescribing Scenarios based on definitions provided in Additional file 1: Table S1

Overall, 3.9% of antibiotic-treated outpatients (Fig. 2a) and 13.2% of inpatients (Fig. 2b) were classified according to Scenario A (confirmed infection), whereas Scenario B (insufficient evidence) was assigned to 2.0 and 6.0%, respectfully. The vast majority of patients did not have sufficient information in their chart to confirm/refute bacterial infection, classified according to Scenario C (94.1% outpatients vs. 80.8% inpatients). Scenario C was most prevalent in Poland (20.9%), with Scenario A highest in Peru (19.4%) and Mexico (14.0%). Scenario D (possible redundant use), was identified for over 40% of Argentinian, Mexican and Peruvian patients, regardless of status, (Fig. 2c), with an overall prevalence of 43.1% in outpatients and 50.4% in inpatients.

Antibiotic use in the population of patients prescribed antibiotics according to Scenario C (total *n* = 17) and Scenario B/C (total *n* = 280) is presented in Table 3. Similar to the findings in the overall population of antibiotic users, β-lactam agents accounted for the majority of antibiotics, by class, prescribed to Scenario C [87.5% of inpatients (*n* = 14/16)], and Scenario B/C populations [outpatients: 81.6% (*n* = 40/49); inpatients: 85.7% (*n* = 198/231)] (Table 3). Clindamycin was the most commonly prescribed Scenario B/C agent for both outpatients (18.4%; *n* = 9/51) and inpatients (42.0%; *n* = 97/231) with cefuroxime the most common inpatient antibiotic under Scenario C (*n* = 5/16; 31.3%) (Additional file 4: Table S4).

**Table 3:** Patients reporting **≥**1 antibiotic by class, and mean duration of antibiotic use, for patients administered antibiotics under Prescribing Scenario C and Scenario B/C- by patient status and country

	Outpatient						Inpatient					
	Argentina	Hungary	Mexico	Peru	Poland	Total	Argentina	Hungary	Mexico	Peru	Poland	Total
Patients prescribed antibiotics according to Scenario C, N	–	1	–	–	–	1	2	2	2	1	9	16
Mean (95% CI) duration of use of antibiotics, Scenario C, days	–	7.0 (3.3, 14.7)	–	–	–	7.0 (3.3, 14.7)	6.0 (3.4,10.6)	5.5 (3.0, 9.9)	5.5 (3.0, 9.9)	5.0 (2.1, 12.0)	5.4 (4.1, 7.2)	5.5 (4.2, 7.2)
Patients prescribed ≥1 antibiotic, by class, Scenario C, n (%) ^a,c^												
*β-lactam agent*	–	1 (100.0)	–	–	–	1 (100.0)	1 (50.0)	2 (100.0)	2 (100.0)	1 (100.0)	8 (88.9)	14 (87.5)
*Aminoglycide*	–	–	–	–	–	–	1 (50.0)	–	1 (50.0)	–	–	2 (12.5)
*Lincosamide*	–	–	–	–	–	–	1 (50.0)	1 (50.0)	1 (50.0)	–	–	3 (18.8)
*Macrolide*	–	–	–	–	–	–	–	–	–	–	1 (11.1)	1 (6.3)
Patients prescribed antibiotics according to Scenario B/C, N	16	2	6	17	8	49	50	40	49	54	38	231
Mean (95% CI) duration of use of antibiotics, Scenario B/C, days	9.5 (8.0, 11.2)	7.0 (4.1, 11.8)	8.2 (6.2, 10.8)	6.5 (5.4, 7.8)	8.5 (6.7,10.8)	7.9 (6.8, 9.0)	8.9 (8.1, 9.8)	7.2 (6.4,8.1)	15.8 (14.8, 17.0)	14.9 (13.9, 16.0)	8.1 (7.3, 9.1)	10.4 (10.0, 10.9)
Patients prescribed ≥1 antibiotic, by class, under Scenario B/C n (%)^b,c^												
*β-lactam agent*	15 (93.8)	2 (100.0)	4 (66.7)	12 (70.6)	7 (87.5)	40 (81.6)	28 (56.0)	38 (95.0)	44 (89.8)	54 (100.0)	34 (84.5)	198 (85.7)
*Lincosamide*	6 (37.5)	–	3 (50.0)	–	–	9 (18.4)	35 (70.0)	6 (15.0)	29 (59.2)	24 (44.4)	3 (7.9)	97(42.0)
*Aminoglycide*	–	–	–	1 (5.9)	–	1 (100.0)	5 (10.0)	6 (15.0)	5 (10.2)	1 (1.9)	1 (2.6)	18 (7.8)
*Glycopeptide*	–	–	–	–	–	–	2 (4.0)	–	8 (16.3)	4 (7.4)	1 (2.6)	15 (6.5)
*Other*	6 (37.5)	–	1 (16.7)	5 (29.4)	1 (12.5)	13 (26.5)	11 (22.0)	–	3 (6.1)	1 (1.9)	1 (2.6)	15 (6.5)
*Macrolide*	–	–	–	1 (5.9)	1 (12.5)	2 (4.1)	–	–	1 (2.0)	–	3 (7.9)	4 (17.3)
*Fluoroquinolone*	–	–	–	–	–	–	–	1 (2.5)	2 (4.0)	–	1 (2.6)	4 (1.7)

*CI* Confidence Interval

^a^ Among population of patients under prescribing scenario C

^b^ Among population of patients under prescribing scenario B + C

^c^ Reported for classes of antibiotics administered to > 1.0% of the total inpatient and/or outpatient population

Overall mean (95% CI) duration of use of antibiotics prescribed according to Scenario C was 7.0 (3.3, 14.7) days and 5.5 (4.2, 7.2) days for outpatients and inpatients, respectively; for Scenario B/C classified antibiotics, duration was 7.9 (6.8, 9.0) days in outpatients, and 10.4 (10.0, 10.9) days in inpatients (Table 3).

## Discussion

The results of this study demonstrate that, among pediatric varicella cases in the 5 studied countries, rates of infectious complications in outpatient (12.2%) and inpatient (78.8%) settings are considerable, with approximately 33% of all inpatient complications involving a bacterial infection, of which > 15% were confirmed microbiologically. Consequently, almost 70% of the inpatient cohort, and an additional 12.7% of outpatients, were prescribed ≥1 antibiotic agent for the treatment of varicella-related complications, for which skin and soft tissue infections were predominant.

It is estimated that between 37 and 78% of all-cause pediatric hospital visits worldwide, and 20% of pediatric ambulatory care setting consultations, result in an antibiotic prescription [14–16]. This not only makes antibiotics the highest prescribed drugs in hospital settings [16], but also predisposes to inappropriate prescribing practices, both with respect to the treatment of non-bacterial infections, as well as the misuse of broad spectrum antibiotics, contributing to antibiotic resistance [15, 16].

The top five most commonly prescribed antibiotic agents, in this pooled population, were clindamycin, ceftriaxone, cefuroxime, penicillin, and cefalexin, with β-lactam agents consistently identified as the most frequently prescribed class. These findings are consistent with the Worldwide Antibiotic Resistance and Prescribing in European Children (ARPEC) point prevalence survey, which ascertained inpatient pediatric antibiotic prescribing patterns across 41 countries [14]. Results of ARPEC found β-lactam agents to account for over 50% of all antibiotic prescriptions across all regions, except North America. In addition, by agent, Eastern European estimates for antibiotic use, which encompasses both Poland and Hungary, confirm ceftriaxone as the most prescribed antibiotic within this region; Latin American antibiotic use included ceftriaxone and clindamycin within the top four below meropenem (1st) and vancomycin (2nd) [14].

As the initial study design did not allow for a definitive link to be made between complication and antibiotic, an exact post-hoc classification of antibiotic use was not possible. Instead, to approximate antibiotic prescribing patterns, a set of conservative assumptions were implemented, guiding patient classification into four prescribing scenarios. Consequently, the majority of patients were administered antibiotics without sufficient data in their patient chart to confirm/refute microbiological confirmation (Scenario B), accounting for just over 80% of inpatients and 94.1% of outpatients. Patients under Scenario C (approximation to ‘inappropriate use’) accounted for 6% of inpatients and 2% of outpatients. Recent studies report between 33 and 50% of in-hospital pediatric antibiotic prescriptions are deemed inappropriate [17–19], with a U. S pediatric primary care setting reporting 30% [20]. The rates reported in the present study likely underrepresent the true magnitude of inappropriate and appropriate prescribing practices, the latter represented by patients under Scenario A (3.9% of outpatients; 13.2% of inpatients). In interpreting the rates of possibly inappropriate antibiotic use, one needs to take into consideration that for skin and soft tissue infections, the most frequent complications described here, bacteriological isolation is difficult, and the diagnosis is mainly clinical; furthermore, microbiological evidence suggests that complicated skin and soft tissue infections are predominantly associated with Gram-positive bacteria for which antibiotic treatment is indicated [21]. Similarly, for pneumonia which may be caused by bacteria whose isolation is difficult, diagnosis is mainly based on clinical, laboratory or radiographic evidence which are often sufficient for antibiotic treatment.

Although the majority of children managed in an outpatient setting did not require antibiotic treatment, antibiotic use in the 5 studied countries was high, and the annual estimated number of cumulative days of antibiotic use was found to range from approximately 80,000 days in Hungary to over 2.5 million days in Argentina. Antimicrobial resistance (AMR) a serious worldwide health problem, has the potential to transform common pathogens into dangerous infections [22]. Latin America reports elevated rates of AMR for pathogens involved in respiratory tract infection, such as *S. pneumoniae* [23, 24]. This is in addition to the extremely high burden of multi-drug resistance tuberculosis in Peru [25]. Avoidance of additional antibiotic burden warrants consideration, particularly if vaccine-preventable. In fact, vaccines that can prevent bacterial infections, or viral infections that can result in bacterial complications, represent a potential tool in antimicrobial stewardship programmes, fighting resistance by limiting spread of disease. This is exemplified by pneumococcal conjugate vaccines, which contributed to the decline of invasive pneumococcal disease in Europe, as well as rates of AMR [26].

The burden of varicella may also be addressed through immunization programs. Monovalent or combination (measles, mumps, rubella, and varicella; MMRV) varicella- containing vaccines are approved in immunocompetent children ≥12 months of age in each of the five countries studied [27]; Argentina introduced universal varicella vaccination in 2015, Peru in 2018, and Hungary announced their intention to introduce universal varicella vaccination starting in 2019. Varicella vaccines have been consistently shown in clinical trials and observational studies to be well tolerated and effective in preventing disease transmission [28]. Notwithstanding, only about 40 countries around the world have implemented universal varicella vaccination programmes [29]. In countries adopting universal varicella vaccination, dramatic declines in varicella incidence and associated morbidity and mortality have been reported [28, 30].

The current findings should be interpreted in consideration of the study limitations. A major limitation of the current study, as discussed above, relates to the fact that the post-hoc study design prevented a definitive link between antibiotic use and type of complication. Consequently, definitive “use classification” could not be assigned, and the assumptions implemented may have resulted in over or underestimation of rates across different prescribing scenarios. A further limitation is related to the assessment of antibiotic use by class and agent: as prescribing patterns are subject to regional variations and differences in clinical practice guidelines, the rates observed for the total inpatient and outpatient populations may be skewed due to inconsistencies in prescribing practices across countries. Furthermore, the slightly higher number of outpatients in Peru may have skewed the results for the total outpatient population towards the Peruvian data. The lack of information about the type of pathogens and antibiotic susceptibility is another constraint. Finally, the retrospective chart review design of the individual studies contributes to 1) a selection bias due to an overrepresentation of care-seekers, potentially including a subset of patients with more severe disease, 2) an information bias, as data extracted from medical charts is subject to missing, inconsistent or erroneous information, and human error during transcription.

## Conclusions

Regardless of limitations, the reported high rates of infectious complications and antibiotic use are indicative of high varicella-associated patient, caretaker, and healthcare burden [8, 11] as well as increased risk for antibiotic resistance, both of which could be averted through universal varicella vaccination; whereas the low rates of documented microbiological confirmation possibly suggest considerable antibiotic misuse for management of varicella complications. Antimicrobial stewardship programmes should be coupled with treatment modalities that prevent the spread of infectious diseases which either directly or indirectly necessitate antibiotic treatment.

## Additional files

Additional file 1: **Table S1.** Definitions of Prescribing Scenarios by Complication Profile. (DOCX 16 kb)

Additional file 2: **Table S2.** Patients reporting ≥1 antibiotic by agent and mean duration of antibiotic use, among antibiotic users by patient status and country (DOCX 24 kb)

Additional file 3: **Table S3.** Cumulative Days of Antibiotic Use by Country based on Annual Number of Pediatric Varicella Cases (DOCX 21 kb)

Additional file 4: **Table S4.** Patients reporting ≥1 antibiotic by agent, and mean duration of antibiotic use, for patients administered antibiotics under Prescribing Scenario C and Scenario B/C- by patient status and country (DOCX 20 kb)

## Data Availability

The datasets generated and/or analysed during the current study are not publicly available due to ethical reasons, to ensure the privacy of the patient level data utilized in the current study but are available from the corresponding author on reasonable request.

## Abbreviations

AMR: antimicrobial resistance
ARPEC: Worldwide Antibiotic Resistance and Prescribing in European Children
CI: confidence interval
SD: standard deviation
UVV: universal varicella vaccination
VZV: varicella zoster virus
