# Supplementary material for: The use of antibiotics in the treatment of pediatric varicella patients: real-world evidence from the multi-country MARVEL study in Latin America & Europe
Source: BMC Public Health. 2019 Jun 26;19:826. doi: 10.1186/s12889-019-7071-z (PMC6595594; doi:10.1186/s12889-019-7071-z)
Supplement: Supplementary file 1 — Table S1. Definitions of Prescribing Scenarios by Complication Profile. (DOCX 16 kb) [file 12889_2019_7071_MOESM1_ESM.docx]

Additional file 1: Table S1. Definitions of Prescribing Scenarios by Complication Profile

| **Complication Profile** | | | **Prescribing Scenario*** | **Prescribing Scenario Definition** |
| --- | --- | --- | --- | --- |
| **Type of complication Indicated?** | **Microbiologically confirmed?** | **Type of infection indicated?** |  |  |
| Infectious | yes | Bacterial | A | Evidence of bacterial infection in chart |
| Infectious | yes | Non-bacterial (viral, fungal, or “other” specified) | B | Insufficient evidence to confirm/refute bacterial infection in chart |
| Infectious | no or N/A or yes | Missing | C | Absence of evidence to confirm bacterial infection in chart |
| Infectious | no or N/A | Bacterial | C | Absence of evidence to confirm bacterial infection in chart |
| Non-Infectious | - | | B | Insufficient evidence to confirm/refute bacterial infection in chart |
| Missing | Patients who were prescribed an antibiotic without any mention of a complication | | C | Absence of evidence to confirm bacterial infection in chart |
| *Scenario D (potentially redundant antibiotic use) is defined as number of antibiotics prescribed greater than the number of infectious complications. | | | | |
