# Supplementary material for: The use of antibiotics in the treatment of pediatric varicella patients: real-world evidence from the multi-country MARVEL study in Latin America & Europe
Source: BMC Public Health. 2019 Jun 26;19:826. doi: 10.1186/s12889-019-7071-z (PMC6595594; doi:10.1186/s12889-019-7071-z)
Supplement: Supplementary file 2 — Table S2. Patients reporting ≥1 antibiotic by agent and mean duration of antibiotic use, among antibiotic users by patient status and country (DOCX 24 kb) [file 12889_2019_7071_MOESM2_ESM.docx]

Additional file 2: Table S2. Patients reporting ≥1 antibiotic by agent and mean duration of antibiotic use, among antibiotic users by patient status and country

^**^

|  | Outpatient | | | | | | Inpatient | | | | | |
| --- | --- | --- | --- | --- | --- | --- | --- | --- | --- | --- | --- | --- |
|  | Argentina  N=75 | Hungary  N=75 | Mexico  N=75 | Peru  N=101 | Poland  N=75 | Total  N=401 | Argentina  N=75 | Hungary  N=81 | Mexico  N=77 | Peru  N=78 | Poland  N=75 | Total  N=386 |
| Patients prescribed ≥1 antibiotic, n (%) | 17 (22.7) | 2 (2.7) | 6 (8.0) | 17 (16.8) | 9 (12.0) | 51 (12.7) | 54 (72.0) | 45 (55.6) | 57 (74.0) | 67 (85.9) | 43 (57.3) | 266 (68.9) |
| Mean (95% CI), number of antibiotics prescribed per patient ^*^ | 1.8  (1.2, 2.5) | 1.0  (0.2, 3.1) | 1.5  (0.7, 2.7) | 1.1  (0.7, 1.7) | 1.1  (0.6, 1.9) | 1.4  (1.1, 1.7) | 2.1  (1.7, 2.5) | 1.7  (1.4, 2.1) | 2.5  (2.1, 2.9) | 2.2  (1.8, 2.5) | 1.5  (1.2, 1.9) | 2.0  (1.9, 2.2) |
| Mean (95% CI) duration of antibiotics prescribed per patient, days ^*^ | 9.5  (8.1, 11.1) | 7.0  (4.1, 11.8) | 8.2  (6.2, 10.8)) | 6.5  (5.3, 7.8) | 8.7  (6.9, 10.7) | 7.9  (6.9, 10.5) | 10.6  (9.7, 11.5) | 7.2  (6.4, 8.0) | 16.1  (15.0, 17.1) | 14.6  (13.7, 15.5) | 8.6  (7.7, 9.5) | 10.9  (10.5, 11.3) |
| Patients prescribed ≥1 antibiotic, by agent, n (%) ^*, §, ‡^ | | | | | | | | | | | | |
| *Clindamycin* | 7 (41.2) | - | 3 (50.0) | - | - | 10 (19.6) | 39 (72.2) | 8 (17.8) | 35 (61.4) | 31 (46.3) | 4 (9.3) | 117 (44.0) |
| *Ceftriaxone* | 6 (35.3) | - | 1 (16.7) | - | - | 7 (13.7) | 12 (22.2) | 1 (2.2) | 20 (35.1) | 6 (9.0) | 6 (14.0) | 45 (16.9) |
| *Cefuroxime* | - | - | - | - | 2 (22.2) | 2 (3.9) | 3 (5.6) | 11 (24.4) | 3 (5.3) | 2 (3.0) | 23 (53.5) | 42 (15.8) |
| *Penicillin* | - | 2 (100.0) | 1 (16.7) | 3 (17.6) | - | 6 (11.8) | 5 (9.3) | 7 (15.6) | 11 (19.3) | 14 (20.9) | 2 (4.7) | 39 (14.7) |
| *Cephalexin* | 5 (29.4) | - | - | 1 (5.9) | - | 6 (11.8) | 6 (11.1) | 2 (4.4) | 1 (1.8) | 30 (44.8) | - | 39 (14.7) |
| Mean (95% CI) duration of use, by agent, days ^*, §^ | | | | | | | | | | | | |
| *Clindamycin* | 4.9  (3.5, 6.8) | - | 6.0  (3.8, 9.5) | - | - | 5.4  (4.1, 7.2) | 5.4  (4.7, 6.2) | 4.0  (2.8, 5.7) | 8.1  (7.2, 9.1) | 6.8  (5.9, 7.8) | 8.5  (6.1, 11.9) | 6.3  (5.7, 7.0) |
| *Ceftriaxone* | 3.3  (2.2, 5.2) | - | 1.0  (0.1, 7.1) | - | - | 1.8  (0.7, 4.9) | 5.6  (4.4, 7.1) | 4.0  (1.5, 10.7) | 6.4  (5.3, 7.6) | 7.0  (5.2, 9.4) | 8.5  (6.5, 11.2) | 6.1  (4.9, 7.6) |
| *Cefuroxime* | - | - | - | - | 5.0  (2.7, 9.3) | 5.0  (2.7, 9.3) | 8.0  (5.4, 11.9) | 5.0  (3.8, 6.5) | 3.3  (1.8, 6.2) | 5.5  (3.0, 9.9) | 5.5  (4.6, 6.5) | 5.3  (4.3, 6.4) |
| *Penicillin* | - | 7.0  (4.1, 11.8) | 1.0  (0.1, 7.1) | 7.0  (4.6, 7.1) | - | 3.7  (1.8, 7.3) | 5.6  (3.9, 8.1) | 4.9  (3.5, 6.8) | 6.2  (4.9, 7.8) | 7.4  (6.1, 8.9) | 11.0  (7.2, 16.7) | 6.7  (5.8, 7.7 |
| *Cephalexin* | 7.2  (5.2, 10.0) | - | - | 7.0  (3.3, 14.7) | - | 7.1  (4.7, 10.6) | 6.3  (4.6, 8.7) | 1.5  (0.5, 4.7) | 7.0  (3.3, 14.7) | 6.3  (5.4, 7.2) | - | 4.5  (3.2, 6.4) |
| CI = Confidence Interval  * Among population of antibiotic users  ^†^ Reported for classes of antibiotics administered to >1.0% of the total inpatient and/or outpatient population  ^‡^ Patients may have been prescribed ≥1 antibiotic  ^§^ Overall Top 5 antibiotic agents prescribed are reported | | | | | | | | | | | | |
