# Supplementary material for: The use of antibiotics in the treatment of pediatric varicella patients: real-world evidence from the multi-country MARVEL study in Latin America & Europe
Source: BMC Public Health. 2019 Jun 26;19:826. doi: 10.1186/s12889-019-7071-z (PMC6595594; doi:10.1186/s12889-019-7071-z)
Supplement: Supplementary file 3 — Table S3. Cumulative Days of Antibiotic Use by Country based on Annual Number of Pediatric Varicella Cases (DOCX 21 kb) [file 12889_2019_7071_MOESM3_ESM.docx]

Additional file 3: Table S3. Cumulative Days of Antibiotic Use by Country based on Annual Number of Pediatric Varicella Cases

|  | **Country** | | | | |
| --- | --- | --- | --- | --- | --- |
| **Parameter** | **Argentina** | **Hungary** | **Mexico** | **Peru** | **Poland** |
| **Annual pediatric outpatient antibiotic use** |  |  |  |  |  |
| *Estimated annual number of pediatric varicella outpatients ^†^* | 1,222,961 | 374,062 | 207,320.95 | 1,288,924 | 1,717,685 |
| *Proportion of pediatric outpatients who receive antibiotics ^‡^* | 22.7% | 2.7% | 8.0% | 16.8% | 12.0% |
| *Mean number of days of antibiotic use per pediatric outpatient ^‡^* | 9.5 | 7 | 8.2 | 6.5 | 8.7 |
| *Cumulative number of days of antibiotic use by pediatric outpatients per year* | 2,637,315 | 70,698 | 136,003 | 1,407,505 | 1,793,263 |
| **Annual pediatric inpatient antibiotic use** |  |  |  |  |  |
| *Estimated annual number of hospitalized pediatric varicella cases ^†^* | 1,951 | 1,792 | 12,718 | 17,204 | 3,505 |
| *Proportion of pediatric inpatients who receive antibiotics ^‡^* | 72.0% | 55.6% | 74.0% | 85.9% | 57.3% |
| *Mean number of days of antibiotic use per pediatric inpatient  ^‡^* | 10.6 | 7.2 | 16.1 | 14.8 | 8.6 |
| *Cumulative number of days of antibiotic use by pediatric inpatients per year* | 14,890 | 7,174 | 151,525 | 218,718 | 17,272 |
| **Cumulative number of days of pediatric antibiotic use per year** | 2,652,205 | 77,871 | 287,528 | 1,626,223 | 1,810,535 |

^†^ Estimates based on data and extrapolations presented in the individual country study reports for Argentina [8], Hungary [12], Mexico [13], Peru [14], Poland [15]

^‡^ Estimates derived from current analysis
