# Supplementary material for: The use of antibiotics in the treatment of pediatric varicella patients: real-world evidence from the multi-country MARVEL study in Latin America & Europe
Source: BMC Public Health. 2019 Jun 26;19:826. doi: 10.1186/s12889-019-7071-z (PMC6595594; doi:10.1186/s12889-019-7071-z)
Supplement: Supplementary file 4 — Table S4. Patients reporting ≥1 antibiotic by agent, and mean duration of antibiotic use, for patients administered antibiotics under Prescribing Scenario C and Scenario B/C- by patient status and country (DOCX 20 kb) [file 12889_2019_7071_MOESM4_ESM.docx]

Additional file 4: Table S4. Patients reporting ≥1 antibiotic by agent, and mean duration of antibiotic use, for patients administered antibiotics under Prescribing Scenario C and Scenario B/C- by patient status and country

|  | Outpatient | | | | | | Inpatient | | | | | |
| --- | --- | --- | --- | --- | --- | --- | --- | --- | --- | --- | --- | --- |
|  | Argentina | Hungary | Mexico | Peru | Poland | Total | Argentina | Hungary | Mexico | Peru | Poland | Total |
| Patients prescribed antibiotics according to Scenario C, N | - | 1 | - | - | - | 1 | 2 | 2 | 2 | 1 | 9 | 16 |
| Mean (95% CI) duration of use of antibiotics, Scenario C, days | - | 7.0  (3.3, 14.7) | - | - | - | 7.0  (3.3, 14.7) | 6.0  (3.4,10.6) | 5.5  (3.0, 9.9) | 5.5  (3.0, 9.9) | 5.0  (2.1, 12.0) | 5.4  (4.1, 7.2) | 5.5  (4.2, 7.2) |
| Patients prescribed ≥1 antibiotic, by agent, Scenario C, n (%)^*, ‡^ |  |  |  |  |  |  |  |  |  |  |  |  |
| *Cefuroxime* | - | - | - | - | - | - |  |  | - | - | 5 (55.6) | 5 (31.3) |
| *Clindamycin* | - | - | - | - | - | - | 1 (50.0) | 1 (50.0) | 1 (50.0) | - |  | 3 (25.0) |
| *Amoxicillin* | - |  | - | - | - |  | - | - | 1 (50.0) |  | 1 (11.1) | 2 (12.5) |
| *Ceftriaxone* | - | - | - | - | - | - | 1 (50.0) |  | - | - | 2 (22.2) | 3 (18.8) |
| *Penicillin* | - | 1 (100.0) | - | - | - | 1 (100.0) | - | - | - | 1 (100.0) | - | 1 (6.3) |
| Patients prescribed antibiotics according to Scenario B/C, N | 16 | 2 | 6 | 17 | 8 | 49 | 50 | 40 | 49 | 54 | 38 | 231 |
| Mean (95% CI) duration of use of antibiotics, Scenario B/C, days | 9.5  (8.0, 11.2) | 7.0  (4.1, 11.8) | 8.2  (6.2, 10.8) | 6.5  (5.4, 7.8) | 8.5  (6.7,10.8) | 7.9  (6.8, 9.0) | 8.9  (8.1, 9.8) | 7.2  (6.4,8.1) | 15.8  (14.8, 17.0) | 14.9  (13.9, 16.0) | 8.1  (7.3, 9.1) | 10.4  (10.0, 10.9) |
| Patients prescribed ≥1 antibiotic, by agent, Scenario B/C, n (%) ^¶, ‡^ |  |  |  |  |  |  |  |  |  |  |  |  |
| *Clindamycin* | 6 (37.5) | - | 3 (50.0) | - | - | 9 (18.4) | 35 (70.0) | 6 (15.0) | 29 (59.2) | 24 (44.4) | 3 (7.9) | 97 (42.0) |
| *Ceftriaxone* | 6 (37.5) | - | 1 (16.7) | - | - | 7 (14.3) | 9 (18.0) | 1 (2.5) | 15 (30.6) | 6 (11.1) | 6 (15.8) | 37 (16.0) |
| *Cefuroxime* | - | - | - | - | 2 (25.0) | 2 (4.1) | 3 (6.0) | 11 (27.5) | 3 (6.1) | 2 (3.7) | 23 (60.5) | 42 (18.2) |
| *Penicillin* | - | 2 (100.0) | 1 (16.7) | 3 (17.6) | - | 6 (12.2) | 4 (8.0) | 4 (10.0) | 9 (18.4) | 13 (24.1) | 2 (5.3) | 32 (13.9) |
| *Cefalexin* | 5 (31.3) | - | - | 1 (5.9) | - | 6 (12.2) | 5 (10.0) | 2 (5.0) | 1 (2.0) | 25 (46.3) | - | 33 (14.3) |
| CI= Confidence Interval  ^*^ Among population of patients under prescribing scenario C  ^¶^ Among population of patients under prescribing scenario B+C  ^‡^ Overall Top 5 antibiotic agents prescribed are reported | | | | | | | | | | | | |
